# A Diagnostic Challenge: Erdheim Chester Disorder

**DOI:** 10.4274/mirt.galenos.2018.72677

**Published:** 2019-03-19

**Authors:** Mairah Razi, Maria Qubtia, Aamna Hassan, Mudassar Hussain, Abdul Hameed

**Affiliations:** 1 Shaukat Khanum Memorial Cancer Hospital and Research Centre, Clinic of Nuclear Medicine, Lahore, Pakistan; 2 Shaukat Khanum Memorial Cancer Hospital and Research Centre, Clinic of Medical Oncology, Lahore, Pakistan; 3 Shaukat Khanum Memorial Cancer Hospital and Research Centre, Clinic of Pathology, Lahore, Pakistan

**Keywords:** Erdheim-Chester disease, non-Langerhans cell histiocytosis, positron emission tomography

## Abstract

Erdheim-Chester disease (ECD) is a rare, multisystemic, idiopathic disease often associated with BRAF V600E mutation. Its diagnosis is typically delayed and challenging due to its variable manifestations. Although it has an indolent course, advanced stages can manifest fulminant behavior with multiple vital organ involvement. It is a class 2a, non-Langerhans cell histiocytosis with characteristic radiological appearance. Whole body imaging might be helpful, particularly, to assess skeletal lesions. Although widespread disease with typical skeletal involvement on imaging can prompt diagnosis, histopathology with immunohistochemistry is required for confirmation. The disease can also manifest itself with a large variety of central nervous system related or orbital symptoms. Cardiac involvement is quite common. We present an interesting image of a patient with ECD who underwent PET/CT. Informed consent of the subject described in this image is waived by the Institutional Review Board.

## Abstract (tr)

Erdheim-Chester hastalığı (ECD), çoğunlukla BRAF V600E mutasyonu ile ilişkili, nadir, multisistemik ve idiyopatik bir bozukluktur. Tipik olarak farklı belirtileri nedeniyle geç ve zor tanı konulur. Her ne kadar sessiz bir klinik seyri olsa da ileri evrelerde multipl vital organ tutulumu ile fulminan seyir gösterebilir. Karakteristik radyolojik özellikleri olan, sınıf 2a, non-Langerhans hücreli histiyositozlardandır. Tüm vücut görüntüleme, özellikle iskelet lezyonlarını göstermek için, yararlı olabilir. Her ne kadar görüntülemede tipik yaygın iskelet tutulumu tanıyı öne sürse de kesin tanı için histopatolojik doğrulama gereklidir. Hastalık aynı zamanda kraniyal ya da orbital farklı bulgularla da ortaya çıkabilir. Kardiyak tutulum sıktır. Bu yayında ECD’nin PET/BT görüntülerini sunmaktayız.

## Figures and Tables

**Figure 1:**
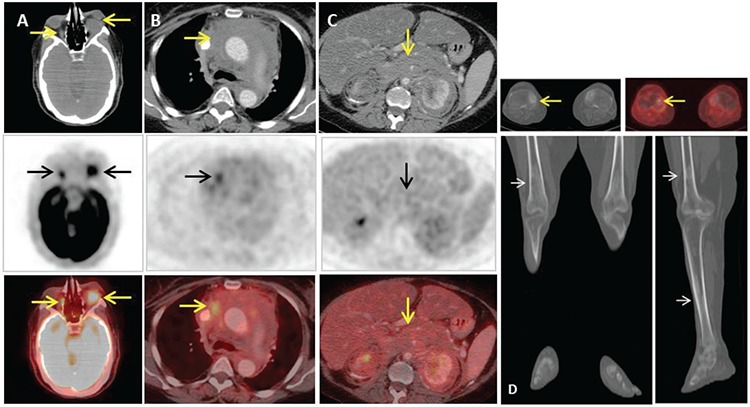
A 59-year-old diabetic, hypertensive, hypothyroid female with cardiac pacemaker for complete heart block, was diagnosed with retroperitoneal fibrosis. Tarsorrhaphy was performed for left eye swelling for corneal/visual protection. Subsequently, she developed renal damage along with lower limb swelling. Baseline non-contrast computed tomography (CT) scan revealed diffuse soft tissue mass around the descending aorta and kidneys, resulting in bilateral hydronephrosis. Follow-up CT scan revealed retroperitoneal mass extending up to the posterior mediastinum. PET/CT was performed with intravenous injection of 10 mCi of ^18^F-FDG. Scan features were suggestive of Erdheim-Chester disease (ECD) in correlation with history and widespread skeletal disease. Axial contrast enhanced PET/CT images (upper row; CT, middle; ^18^F-FDG PET & lower; fusion PET/CT) through orbits (A) showed hypermetabolic intraconal left orbital soft tissue mass (SUV_max_: 6.1) causing proptosis, inseparable from the optic nerve and extraocular muscles. Hypermetabolic thickening of the right optic nerve is also shown. (B) In the mediastinum, diffuse heterogeneously avid infiltrative soft tissue mass (SUV_max_: 4.4) is insinuating between great vessels. (C) Abdominal sections show diffuse retroperitoneal soft tissue mass (SUV_max_: 2.9) encasing branches of the abdominal aorta, infiltrating bilaterally into perinephric space with renal encasement. (D) Coronal and sagittal sections of lower extremities show osteosclerotic changes along long bones. Representative axial PET/CT fusion images show focal avidity overlying sclerosis at medial plateau of the right tibia.

**Figure 2:**
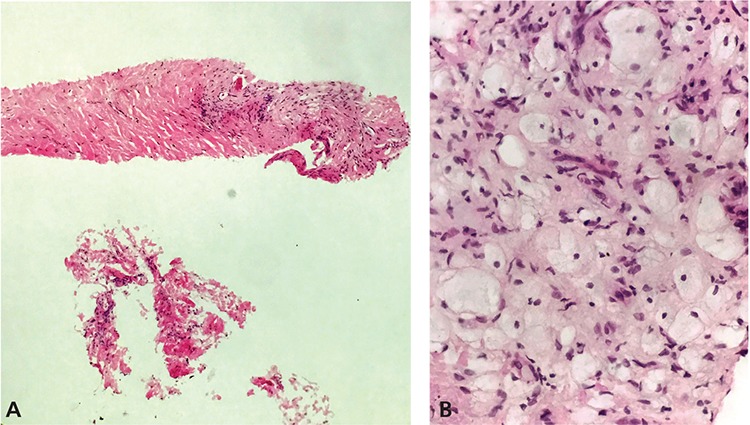
Histopathologic examination of the mediastinal mass revealed dense fibrosis and foamy macrophages. Hematoxylin and eosin (H&E) staining, at 10X and 40X magnifications. Positive CD68 (histiocyte marker) and negative S100 (neural marker), confirming diagnosis of ECD (A; H&E 10X, B; H&E 40X).

**Figure 3:**
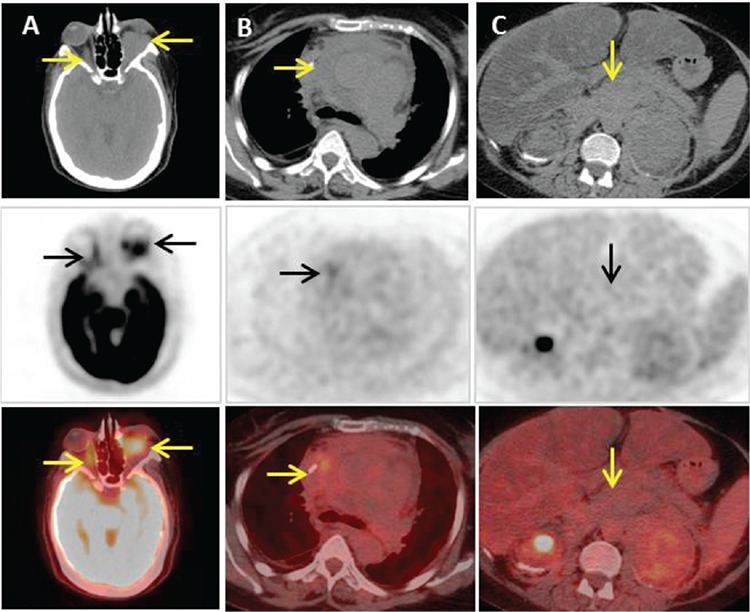
The patient was treated with pegylated interferon-alpha (IFN). Her performance status significantly improved within three weeks along with orbital pain and swelling, pedal edema, and renal functions. Re-evaluation non-contrast PET/CT at six months post IFN initiation; axial CT, PET and fusion images through orbit (A), mediastinum (B) and abdomen (C) demonstrate interval reduction in metabolic activity with stable morphological disease within orbits (SUV_max_: 5.2), mediastinum (SUV_max_: 3.9) and retroperitoneal stations (SUV: 2.4) reflecting stable response. She had good quality of life and tolerated IFN for almost 23 months. Subsequently, she developed cardiac and renal decompensation and died. ECD is a rare chronic disease with delayed presentation, first described by Jakob Erdheim and William Chester as lipid granulomatosis in 1930 (1). Typically, it manifests with characteristic osteosclerosis of diaphysis and metaphysis with epiphyseal sparing of long bones which can be picked up by bone scintigraphy or CT or PET/CT scan (2). Partial involvement of epiphysis has also been reported in the literature (3). ECD indolently involves various organs or fulminant multisystem failure; central nervous system 40-50%, cardiac 75% (4), pulmonary (43%) or pleural involvement (5). Retroperitoneal fibrosis and renal involvement are the commonest presentations (6). ^18^F-FDG PET/CT gained potential importance in early diagnosis of ECD with multisystem involvement enabling whole body acquisition in a single session. Studies have shown excellent specificity of PET scans ranging from 69.2 to 100%; however, sensitivity varies among different organs (range 4.3 to 78.3%) contrary to other imaging modalities (7). PET/CT provides useful information in appreciation of therapy response earlier, depicting metabolic disease activity. One of the recent studies reported effectiveness of PET/CT in management as 48% of cases (8,9). ^18^F-FDG PET scanning depicts metabolic response earlier in neurologic and osseous disease than morphologic changes detected on magnetic resonance imaging (7). Despite characteristic skeletal findings and multisystem involvement, imaging may help in diagnosis, but histologic evaluation is required for confirmation. Our case presents a rare disease in which multidisciplinary approach and appropriate imaging are essential for timely diagnosis and patient management.
